# Effects of COVID-19 Syndemic on Sport Community

**DOI:** 10.3390/jfmk7010019

**Published:** 2022-02-07

**Authors:** Giuseppe Musumeci

**Affiliations:** 1 Department of Biomedical and Biotechnological Sciences, Human, Histology and Movement Science Section, University of Catania, Via S. Sofia n°87, 95123 Catania, Italy; g.musumeci@unict.it; Tel.: +39-095-378-2043; 2 Research Center on Motor Activities (CRAM), University of Catania, Via S. Sofia n°97, 95123 Catania, Italy; 3 Department of Biology, Sbarro Institute for Cancer Research and Molecular Medicine, College of Science and Technology, Temple University, Philadelphia, PA 19122, USA

Nowadays, we live in a society crossed by the greatest public health crisis in over a century: the COVID-19 pandemic. Global governments operated interventions to cut the lines of viral transmission and minimize the spread of the pathogen by imposing lockdowns to the general population and quarantine to positive individuals. However, our experience taught us that managing COVID-19 is not so easy due to the strong interaction between its biological and sociological aspects. Currently the interaction between COVID-19 and noncommunicable diseases such as diabetes, hypertension, obesity, and cardiovascular/respiratory chronic diseases is undeniable, and COVID-19 can be considered a “syndemic” rather than a pandemic [1]. A syndemic refers to a synergistic interplay between biological and socioecological factors, leading to adverse health outcomes. There were three influenza pandemics during the 20th century that caused widespread illness, mortality, social disruption, and significant economic losses, which can be considered syndemic. These occurred in 1918, 1957, and 1968. In each case, mortality rates were determined primarily by five factors: the number of people who became infected, the virulence of the virus causing the pandemic, the speed of global spread, the underlying features and vulnerabilities of the most affected populations, and the effectiveness and timeliness of the prevention and treatment measures that were implemented. The syndemic approach, theorized by Singer in 1990 and extended by Mendenhall in 2012, analyses the health consequences of an illness and its interactions with economic, social, and environmental factors that produce a worsening in the disease outcome. One of the most damaged industries by COVID-19-related restrictions it is the sport one. Sporting events of every size and importance, such as Tokyo 2020 Olympics, were cancelled and postponed due to COVID-19, causing economic losses and generating tensions within the athletes’ community [2]. For example, in Italy, it has been estimated that a lot of people have had to suspend their work due to the forced closure of swimming pools, fitness centres and nonprofessional sports associations. Furthermore, blocking all these sporting activities reduced the possibilities of practicing physical activity for the general population. After more than two years of the COVID-19 syndemic, it has emerged that a sedentary lifestyle, with less than ten minutes of physical activity per week, is correlated with greater odds of getting infected, being admitted to the intensive care unit recovery, or death [3]. The impairments generated by COVID-19 anticontagion restrictions affected individual and team sports athletes worldwide, causing a reduction in training volume, frequency, duration, intensity, and motivation. Remote coaching has been employed to try to maintain adequate performance levels during lockdown, however, this kind of methodology appears to be effective only for elite athletes due to its expensive and difficult-to-access nature. The performance of an athlete is strongly influenced by their mental health status, and the COVID-19 outbreak promoted isolation [4]. The COVID-19 pandemic forced most athletes to cease their regular training activities, with possible consequences on both mental and physical health. It was highlighted by Şenışık et al. [5] that athletes had a better mental health status compared to nonathletes due to the positive effect of the high amount of physical activity performed before the COVID-19-related isolation period. The latter denotes the importance of proper physical activity for mental and physical health. Moreover, among the athletes themselves, those who were able to practice physical activity at high intensity during lockdown reported better mental and physical health [6]. The differences in the economic possibilities of athletes to access various methodologies of training have been exacerbated by the COVID-19 syndemic. Athletes with less financial support had fewer opportunities to maintain good training levels and overall physical health, another example of how COVID-19 did not put everyone on the “same playfield”. Another important aspect to consider is the training specificity. It was nearly impossible, especially for team sport athletes, to perform training sessions with the right amount of sport-specific exercise during the COVID-19 restrictions. This situation, relating to the lack of coaches’ supervision, might increase the possibility of nonoptimal performance and the risk of getting injured for players with the return to play [6]. Furthermore, the political approach of governments amplified the stressful effects of COVID-19 on the sport community. Nonprofessional sports associations and fitness centres were not allowed to reopen as quickly as the professional sports associations, increasing the social tension among people within the sport community. Today, in the first month of 2022, we are still living within the COVID-19 syndemic, approaching this new year with more awareness of how to manage it. The sports community has been wounded economically and socially by COVID-19, however, the spirit that resides in it was strong enough to start the recovery. In fact, in these two years, the Sport Community has been able to remain “united and resolute”, driven by an imperative to maintain relevance and presence among those who practice physical activity. To this end, different strategies have been adopted to encourage sports practice, such as the realization of online fitness classes, the maintenance of coach–athlete virtual interaction, as well as the reorganization of structures and sporting events from amateurism to competitive practice. Governments and institutions are called upon to better support sports organizations so that socioeconomic exhaustion caused by the syndemic does not translate into sports depletion.
